# Exploring intervention components in association with changes in preschool children’s vegetable intake: the BRA-study

**DOI:** 10.1186/s13104-021-05629-1

**Published:** 2021-05-31

**Authors:** Anne Lene Kristiansen, Anne Himberg-Sundet, Mona Bjelland, Nanna Lien, René Holst, Lene Frost Andersen

**Affiliations:** 1 Faculty of Humanities, Sports and Educational Science, Department of Sports, Physical Education and Outdoor Studies, University of South-Eastern Norway, PO Box 235, 3603 Kongsberg, Norway; 2 Department of Nutrition, Institute of Basic Medical Sciences, University of Oslo, Blindern, PO Box 1046, 0317 Oslo, Norway; 3 Department of Biostatistics, Institute of Basic Medical Sciences, University of Oslo, Blindern, PO Box 1122, 0317 Oslo, Norway

**Keywords:** Preschool children, Kindergarten, Intervention, Vegetables, Process evaluation, Norway

## Abstract

**Objective:**

The present study aimed to explore kindergarten staffs’ perceived usefulness of intervention components in association with changes in children’s vegetable intake and vegetables served in the kindergarten. Assessment of the perceived usefulness of intervention components consisted of a paper-based questionnaire for the kindergarten staff assessing usefulness of posters, supplementary material and 1-day inspirational course. Children’s vegetable intake in the kindergarten was assessed by direct observation, while vegetables served was assessed by a 5-day weighted vegetable diary.

**Results:**

Seventy-three kindergartens in two counties in Norway participated (response rate 15%) and parental consent was obtained for 633 children 3–5 years of age at baseline (response rate 39%). Mixed effect models indicated a tendency that posters were associated with increased child vegetable intake (P = 0.062). Surprisingly, a low degree of perceived usefulness of supplementary material was associated with the largest increase in child vegetable intake (P = 0.020). No significant associations between perceived usefulness of intervention components and vegetables served in the kindergarten were found. This study indicated a tendency that posters were associated with increased child vegetable intake; however, this may also be due to synergies between multiple intervention components.

*Trial registration* International Standard Randomized Controlled Trials ISRCTN51962956 (http://www.isrctn.com/ISRCTN51962956). Registered 21 June 2016 (retrospectively registered).

**Supplementary Information:**

The online version contains supplementary material available at 10.1186/s13104-021-05629-1.

## Introduction

Benefits of sufficient intake of fruits and vegetables are recognized in the prevention of non-communicable diseases and all-cause mortality [1–5]. Despite this, consumption in many countries [5], including Norway [6–10] is low. Consequently, increasing consumption is a public health priority [5].

Childcare settings, called kindergartens in Norway, frequently consist of multiple units, called departments. Each department is staffed with one pedagogical leader in addition to two or more assistants, and includes 18 children, either of the same or of mixed age. Meals are either brought from home (lunch box), provided by the kindergarten or a combination. Kindergarten staff are mainly responsible for the foods served at the lunch meal. This is mostly sandwich with spreads, but a hot meal is served about once a week [11]. There are normative national guidelines for food and meals served in the kindergartens [12] and they specify that vegetables and fruit/berries should be included in all meals.

Several interventions have been conducted to understand how to increase vegetable intake in early childhood [13–15]. Results indicate that multicomponent interventions are more successful in increasing vegetable consumption compared to single exposure strategies [13]. Still, knowledge on how to successfully increase vegetable intake is limited. Process evaluations are essential to assist in understanding why or why not an intervention was successful [16–18]. Further, it is increasingly recognized that acceptability is an important part of successful implementation [19]. Previous research among schoolchildren has indicated that vegetable intake was higher among those who valued the intervention the most [20, 21]. We hypothesized that perceived usefulness could be considered as a marker of acceptability of the intervention components. The present study therefore aimed to explore the association between kindergarten staffs’ perceived usefulness of the intervention components and changes in children’s vegetable intake and vegetables served in the kindergarten, from baseline to follow-up 1.

## Main text

### Materials and methods

Study design and subjects have been published elsewhere [22, 23]. Briefly, overall aim of the BRA-study was to improve vegetable intake (primary outcome) among preschool children through changing the food environment and food-related practices in kindergarten and home (secondary outcomes). Child consumption of vegetables and vegetables served in the kindergarten were assessed with regards to frequency, variety and amount of vegetables served and consumed.

Children born in 2010–2011, attending public or private kindergartens in the counties of Vestfold and Buskerud, Norway were included. All regular kindergartens (n = 479) in the counties were invited. Seventy-three kindergartens participated (response rate 15.2%) and parental consent was obtained for 633 children (response rate 38.8%). This study was conducted according to the guidelines laid down in the Declaration of Helsinki, and the Norwegian Center for Research Data approved all procedures involving human subjects.

The intervention and its components have been published elsewhere [24, 25]. Briefly, the intervention components to the kindergartens consisted of a 1-day inspirational course (kitchen practice and theory) in addition to resources like aprons, a vegetable memory game, a hand blender, booklets, posters and brochures. The intervention components were related to the four determinants: availability, accessibility, encouragement and role modelling. The intervention focused on changing these determinants, as implemented in the intervention components. The implementation of the intervention was delivered by the kindergarten staff to the children during the daily meals and additional pedagogical activities [24, 25]. The kindergartens were encouraged to change their practices according to their own action plans using the provided training and resources. Posters were for example made to remind staff to serve vegetables (availability, accessibility) and promote (role model, encourage) vegetable intake by the children.

#### Data collection

There were 313 (49%) children in the intervention group, representing 37 kindergartens and 70 departments. The no-intervention control group continued as normal for the duration of the study and participated only by providing data for the effect analysis.

The research team conducted direct observations to assess children’s vegetable intake at two meals (lunch and an afternoon snack meal) in 1 day in the kindergarten. A 5-day weighted vegetable diary was completed by the kindergarten staff to assess amount of vegetables served in the kindergarten. Data was collected at baseline (spring 2015) and at follow-up 1 (spring 2016) and a description of methods are provided elsewhere [22, 23]. By completing all data collections, kindergartens received a gift card of 2000 NOK (Approx. 223 EUR).

In January 2016, process evaluation was conducted in the intervention group, both in kindergartens and at home. Due to a lack of significant effects upon children’s vegetable consumption in the home setting [25, 26], this paper reports on process evaluation from the kindergarten, including one staff member from each kindergarten/department.

Process evaluation consisted of a paper-based questionnaire including several aspects assessing to what degree the intervention components were perceived useful or not for changing usual vegetable practices in the kindergarten. The present study includes responses to the question “to what degree have the following components been important in getting started with the changes you have been working on?” (i.e., during the intervention period). Perceived usefulness of the components was assessed by four response alternatives: “not at all”, “to a small degree”, “to some degree” and “to a large degree”. Nine intervention components were combined into three groups: posters, supplementary material and 1-day inspirational course (Table 1).

**Table 1:** Kindergarten staff’s perceived usefulness of the given intervention materials in the BRA-study (n = 48)

	Not at all	To a small degree	To some degree	To a large degree
Posters				
A large poster (70 × 100 cm) with photos of vegetables	1 (2%)	7 (15%)	17 (35%)	23 (48%)
Two small posters (A4) on amounts of vegetables	2 (4%)	10 (21%)	20 (42%)	16 (33%)
Four small posters (A4) with ideas of “what to do” for each of the four determinants^a^	4 (8%)	13 (27%)	19 (40%)	12 (25%)
Supplementary material				
Four child-aprons	5 (10%)	11 (23%)	13 (27%)	19 (40%)
One fruit and vegetable memory game	2 (4%)	12 (25%)	18 (38%)	16 (33%)
One hand blender	5 (10%)	11 (23%)	16 (33%)	16 (33%)
1-day inspirational course				
Practical training in the kitchen	2 (4%)	7 (15%)	24 (50%)	15 (31%)
A theoretical session	4 (8%)	8 (17%)	25 (52%)	11 (23%)
Make action plans	4 (8%)	8 (17%)	27 (56%)	9 (19%)

Kindergarten departments

^a^Availability, accessibility, role model and encouragement

#### Data analysis

Children’s observed vegetable intake (grams/day) and served vegetables in the kindergarten (grams/day) were dependent variables in separate mixed effects models. For children’s observed vegetable intake, kindergarten, department and participant were used as random effects, while for vegetables served in the kindergarten, kindergarten and department were used as random effects. Time (baseline and follow-up 1) and perceived usefulness were used as fixed effects. The reference category for perceived usefulness was “not at all/to a small degree”. To avoid small subgroups, response alternatives “not at all” and “to a small degree” were merged into one category in analysis. We defined the effect of a component, as the difference in change of vegetable intake from baseline to follow-up 1 between the response alternative “to some degree”/“to a large degree” and the reference category. These effects were estimated by the interaction terms between the intervention components and time (Table 2). The perceived usefulness factor thus measured the effect of being in one of the two other categories relative to being in the reference category. A potential difference in effects of the perceived usefulness categories between the two time points may be attributed to the intervention. It was of interest to assess if the intervention provided the same change in effect of “to some degree” and “to a large degree” levels. This was accommodated by an interaction effect between time and perceived usefulness. Test for significance was done by likelihood ratio tests. Corresponding models were considered for each of the two outcomes and each of the three groups of intervention components. Models including children’s vegetable intake were adjusted for covariates: maternal education, child gender and child year of birth. Participants were included in analyses if they had data on baseline and/or follow-up 1 and if they had data on all covariates. All analyses were conducted using Stata Statistical Software: Release 15. College Station, TX: StataCorp LLC. P values less than 0.05 were considered statistically significant.

**Table 2:** Estimated mean vegetable intake (gram) by component and time in the BRA-study

	Baseline mean (SD)	Follow-up 1 mean (SD)	Effect size (CI 95%)^a^	Likelihood ratio test P
Children’s vegetable intake (n = 161)^b^				
Posters				
Not at all/to a small degree	43.6 (16.5)	69.5 (16.9)		0.062
To some degree	47.2 (12.7)	85.3 (12.9)	12.2 (− 17.8, 42.2)	
To a large degree	35.8 (15.5)	99.4 (15.4)	37.7 (4.6, 70.7)	
Supplementary material				
Not at all/to a small degree	33.0 (14.8)	97.3 (15.0)		0.020
To some degree	48.6 (13.3)	74.1 (13.4)	− 38.8 (− 65.5, − 12.1)	
To a large degree	43.7 (15.1)	86.8 (15.2)	− 21.2 (− 51.1, 8.7)	
1-day inspirational course				
Not at all/to a small degree	37.6 (16.3)	87.7 (16.2)		0.746
To some degree	49.8 (12.8)	99.9 (17.3)	− 11.2 (− 39.8, 17.4)	
To a large degree	33.9 (15.5)	84.1 (19.4)	− 6.7 (− 39.4, 26.0)	
Vegetable served in the kindergarten (n = 34)^c^				
Posters				
Not at all/to a small degree	54.1 (8.6)	56.5 (9.3)		0.243
To some degree	50.5 (5.5)	62.8 (6.3)	10.0 (− 16.1, 36.1)	
To a large degree	42.0 (7.6)	69.4 (8.6)	24.9 (− 4.4, 54.2)	
Supplementary material				
Not at all/to a small degree	45.8 (6.9)	57.2 (6.9)		0.856
To some degree	54.3 (6.6)	69.8 (8.0)	4.2 (− 21.0, 29.3)	
To a large degree	45.6 (7.2)	64.3 (8.6)	7.3 (− 18.7, 33.4)	
1-day inspirational course				
Not at all/to a small degree	49.6 (8.0)	49.4 (8.5)		0.257
To some degree	46.2 (6.0)	65.7 (6.8)	19.6 (− 5.1, 44.3)	
To a large degree	52.0 (6.8)	71.6 (7.9)	19.9 (− 6.3, 46.0)	

The “Effect Size” measures the difference in change from baseline to follow-up, between the given group and the reference group “not at all/to a small degree” extracted from the models as the interactions between intervention component and time

^a^Interaction between intervention component and time

^b^Adjusted for time, maternal education, child gender and child year of birth

^c^Adjusted for time

### Results

Of the 48 departments (69%) that completed the process evaluation, 34 departments (49%) had complete data on process evaluation and the 5-day weighted vegetable diary at baseline and/or at follow-up 1. Of the 217 children that had been observed at baseline and/or at follow-up 1, 161 children (74%) had data on all covariates and the process evaluation.

Analyses indicated a tendency that in kindergartens where posters were considered useful by the staff, an increase in children’s vegetable intake (P = 0.062) was observed (Table 2). Kindergartens who perceived posters useful “to a large degree” showed an additional increase in children’s vegetable intake of 38 g/day (95% CI 4.6, 70.7) compared to those who perceived posters as “not at all/to a small degree” useful.

Interestingly, in kindergartens where staff reported that supplementary material were perceived as “to some degree” useful, a significantly smaller increase in children’s vegetable intake was observed compared to those who reported perceived usefulness to be “not at all/to a small degree”. A mean difference in children’s vegetable intake of 39 g/day between groups was estimated (95% CI − 65.5, − 12.1) (P = 0.020) (Table 2).

No significant effects were found for the perceived usefulness of the 1-day inspirational course upon children’s vegetable intake (Table 2). Furthermore, no significant effects on amount of vegetables served in the kindergarten setting were found for posters, supplementary material or for the 1-day inspirational course (Table 2).

**Fig. 1:**
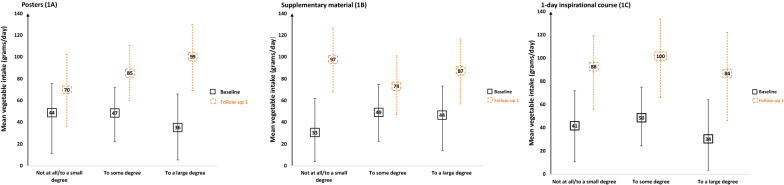
Estimated marginal means of the BRA-study, showing children’s vegetable intake (n = 161) at baseline and at follow-up 1, adjusted for time, maternal education, child gender and child year of birth in relation to what degree the intervention components (posters, supplementary material and 1-day inspirational course) were perceived useful by the kindergarten staff. (Black box) Baseline data, (Orange dotted box) Follow-up 1 data

To assess mean response in the intervention components across time, marginal means were estimated. There seemed to be a trend towards larger increase in children’s vegetable intake with increasing degree of perceived usefulness of posters [“to some degree” (P = 0.03) and “to a large degree” (P < 0.001)] (Fig. 1A).

For supplementary material, children’s vegetable intake increased by 64 g/day among those who reported perceived usefulness to be “not at all/to a small degree” (P < 0.001) while the increase was 43 g/day for those who reported perceived usefulness to be “to a large degree” (P = 0.002) (Fig. 1B).

For 1-day inspirational course upon children’s vegetable intake, a significant increase of 46–50 g/day were seen in all groups [“not at all/to a small degree” (P = 0.001), “to some degree” (P = 0.018) and “to a large degree” (P < 0.001)] (Fig. 1C).

A significant increase in vegetables served were seen for those who reported perceived usefulness of posters to be “to a large degree” (P = 0.024) (Additional file 1: Figure S1A). No significant increases were observed for supplementary material and 1-day inspirational course (Additional file 1: Figure S1B, C).

### Discussion

Findings suggest that there was a trend towards larger increase in children’s vegetable intake with increasing degree of perceived usefulness of posters. However, this may also be due to synergies between intervention components. This is in line with previously reported associations for child vegetable intake and appreciation for an intervention [20, 21].

When kindergartens were visited at follow-up 1, the impression was that many of the intervention kindergartens had displayed the posters. Hence, posters might have had a prompting effect [27] and been a reinforcement of the key messages on amount to be served and how to encourage children to taste and eat vegetables. As an illustration, the two posters on amount of vegetables might have been especially important in creating shifts in children’s vegetable intake as they displayed suggestions for children’s vegetable portion sizes. Studies among adults have reported confusion over portion sizes for fruits and vegetables [28, 29]. This might also be the case here, as the adult recommended intake of fruits and vegetables is 500 g/day [30] in Norway, while there is no precise recommendations for amount for young children.

It is difficult to explain why those who reported the lowest degree of perceived usefulness of supplementary material showed a significantly larger increase in children’s vegetable intake compared to those who perceived such components useful “to some degree”. The hand blender and the aprons should encourage staff to involve children in mealtime tasks. However, 55% of Norwegian kindergartens report to include children in those tasks [11] and thus they might not be perceived the most useful of the components included.

## Limitations

### Strengths and limitations

As evidence of how to increase vegetable intake in early childhood is scare, this study sought to increase knowledge within this field. One limitation was that process evaluations were completed in January 2016, while the follow-up assessments of vegetables were assessed between April–June 2016. Hence, there was a variation in length of implementation period. For vegetables served in the kindergarten, only half of the eligible sample had complete data and it is not known to what extent this sample was representative of the participating kindergartens. Moreover, different persons may have filled in the 5-day weighted vegetable diary at baseline and at follow-up 1, leading to impaired consistency between measures of vegetables served in the kindergarten [26].

## Supplementary Information

**Additional file 1: Figure S1.** Estimated marginal means of the BRA-study, showing vegetables served in the kindergarten departments (n = 34) at baseline and follow-up 1, in relation to what degree the intervention components (posters, supplementary material and 1-day inspirational course) were perceived useful by the kindergarten staff. (Black box) Baseline data, (Orange dotted box) Follow-up 1 data.

## Data Availability

Data used and analyzed during the current study will be available from the corresponding author upon request, provided compliance with current legislation for application for data access in Norway.

## Abbreviation

The BRA-study: An acronym for the Norwegian words “Barnehage” (kindergarten), “gRønnsaker” (vegetables) and “fAmilie” (family). The Norwegian word “BRA” means “good”
