# Supplementary material for: Exploring intervention components in association with changes in preschool children’s vegetable intake: the BRA-study
Source: BMC Res Notes. 2021 May 31;14:214. doi: 10.1186/s13104-021-05629-1 (PMC8165990; doi:10.1186/s13104-021-05629-1)
Supplement: Supplementary file 1 — Additional file 1: Figure S1. Estimated marginal means of the BRA-study, showing vegetables served in the kindergarten departments (n = 34) at baseline and follow-up 1, in relation to what degree the intervention components (posters, supplementary material and 1-day inspirational course) were perceived useful by the kindergarten staff. (Black box) Baseline data, (Orange dotted box) Follow-up 1 data. [file 13104_2021_5629_MOESM1_ESM.docx]

**Figure S1.** Estimated marginal means of the BRA-study, showing vegetables served in the kindergarten departments (n = 34) at baseline and follow-up 1, in relation to what degree the intervention components (posters, supplementary material and 1-day inspirational course) were perceived useful by the kindergarten staff.

(Black box) Baseline data, (Orange dotted box) Follow-up 1 data.
